# Supplementary material for: Effect of User Practice on Prosthetic Finger Control With an Intuitive Myoelectric Decoder
Source: Front Neurosci. 2019 Sep 10;13:891. doi: 10.3389/fnins.2019.00891 (PMC6747011; doi:10.3389/fnins.2019.00891)
Supplement: Supplementary file 1 [file Data_Sheet_1.PDF]

Supplementary Material

SUPPLEMENTARY FIGURES

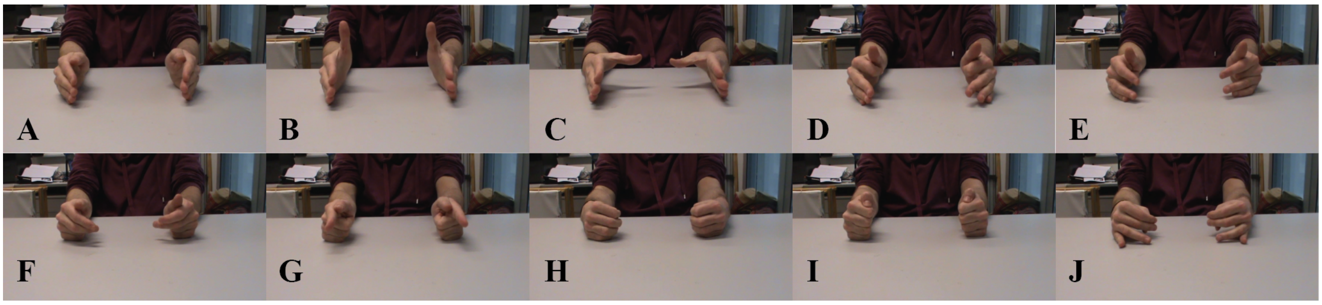

Figure S1. Training exercises. Subjects were instructed to perform the following bilateral mirrored movements: **(A)** rest; **(B)** thumb flexion/extension; **(C)** thumb abduction/adduction; **(D)** index flexion/ extension; **(E)** middle flexion/extension; **(F)** ring/little flexion/extension; **(G)** index pointer; **(H)** cylindrical grip; **(I)** lateral grip; **(J)** tripod grip.

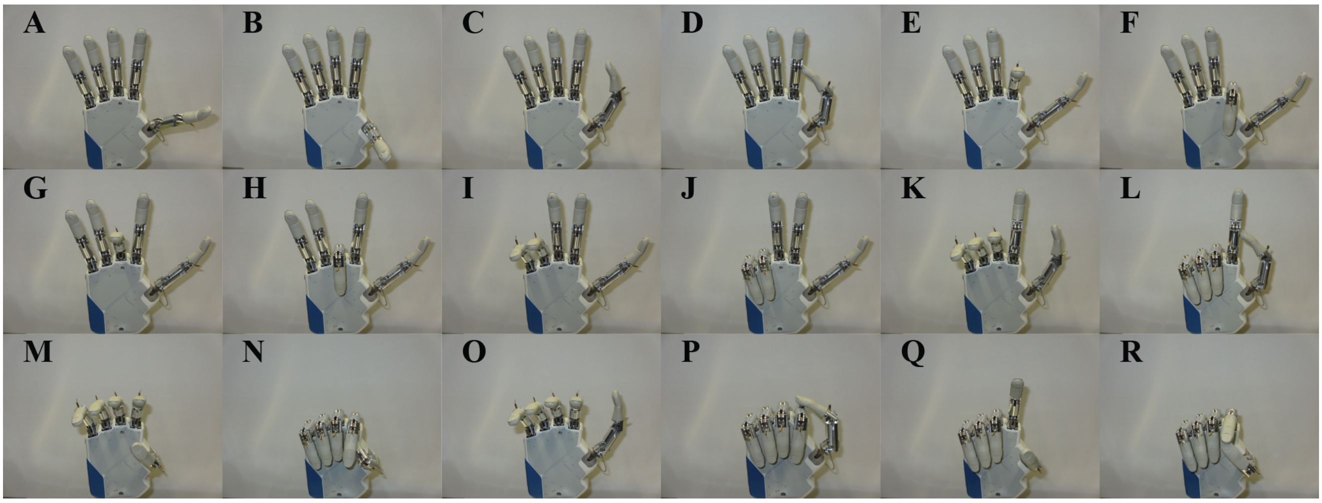

Figure S2. Target poses for real-time posture matching task. **(A)** Thumb abduction (half); **(B)** thumb abduction (full); **(C)** thumb flexion (half); **(D)** thumb flexion (full); **(E)** index flexion (half); **(F)** index flexion (full); **(G)** middle flexion (half); **(H)** middle flexion (full); **(I)** ring/little flexion (half); **(J)** ring/little flexion (full); **(K)** index pointer (half); **(L)** index pointer (full); **(M)** cylindrical grip (half); **(N)** cylindrical grip (full); **(O)** lateral grip (half); **(P)** lateral grip (full); **(Q)** pinch grip (half); **(R)** pinch grip (full).

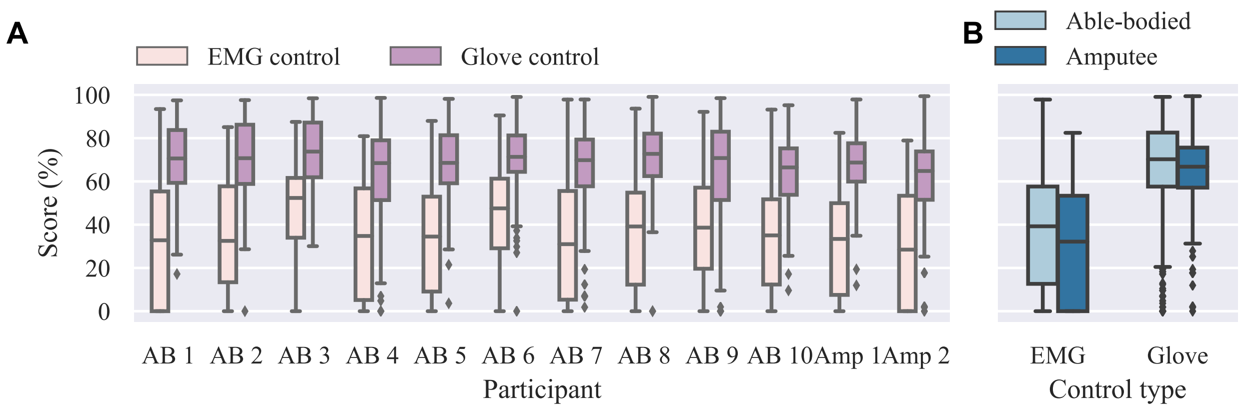

Figure S3. Summary results for real-time experiment (performance scores). **(A)** Performance scores received by participants after trial end shown for individual subjects, separately for EMG and glove control modes; **(B)** summary scores for able-bodied and amputee groups for the two control modes. Straight lines, medians; solid boxes, interquartile ranges; whiskers, overall ranges of non-outlier data; diamonds, outliers; AB, able-bodied; Amp, amputee.

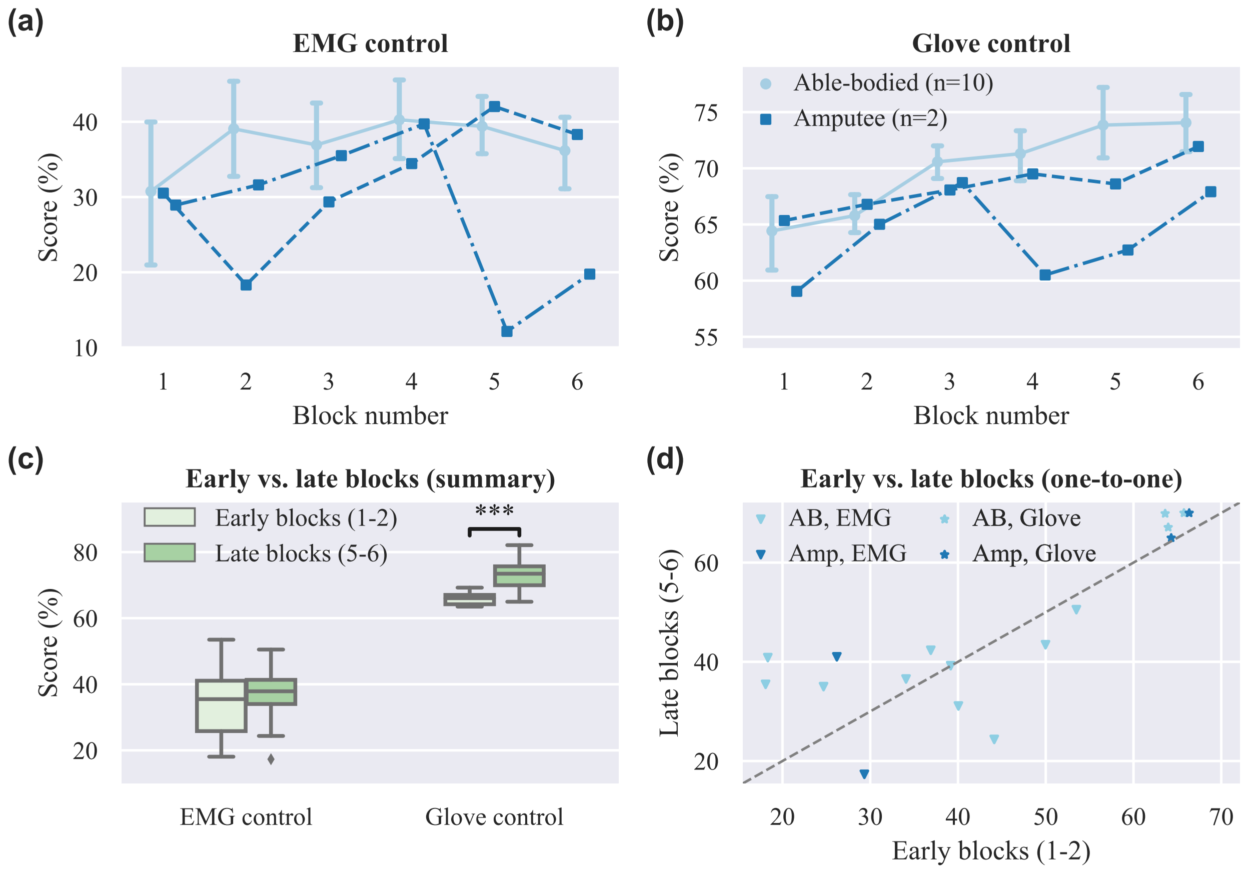

Figure S4. Motor learning curves for real-time experiment. **(A)**-**(B)** Performance scores received by participants after trial end are plotted against the experimental block number for **(A)** electromyography (EMG) and **(B)** glove control; **(C)** comparison of early versus late blocks for grouped participants (able-bodied and amputee); **(D)** one-to-one comparisons of early versus late blocks averages for all participants. Points, means; error bars, 95% confidence intervals estimated via bootstrapping (1000 iterations); triple asterisk, $p < 0.001$.

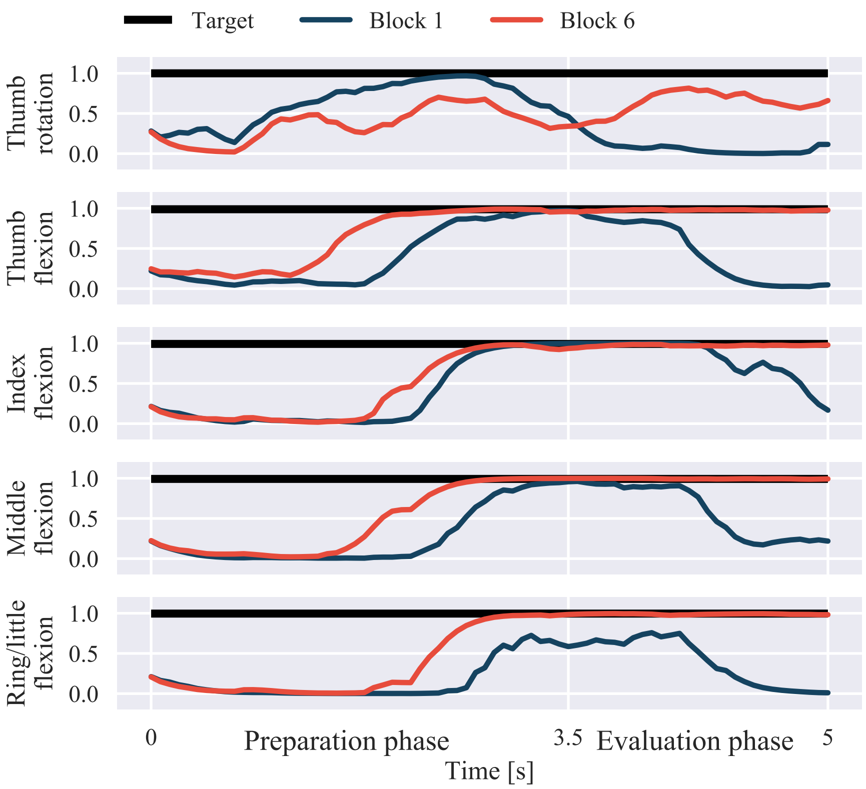

Figure S5. Example of learning effect on myoelectric control performance. Traces of target and actual digit positions of the robotic hand are shown for an able-bodied participant in blocks 1 and 6 of the experiment. The shown traces correspond to the full cylindrical grip (Supplementary Fig. S2N). The received performance scores at the end of the trials were 9.59% and 84.32% for blocks 1 and 6, respectively.

SUPPLEMENTARY METHODS

Mapping CyberGlove II measurements to degrees of actuation of the IH2 Azzurra hand

A linear mapping between the measurements of the 18-degree of freedom (DOF) CyberGlove II (CyberGlove Systems LLC) and the degrees of actuation (DOAs) of the IH2 Azzurra hand (Prensilia s.r.l.) was used. Due to cross-coupling between the data glove sensors Steffen et al. (2011); Wang and Neff (2013), the mapping was identified empirically and its quality was subsequently verified during pilot experiments involving teleoperating the robotic hand in real-time using the data glove.

Let $\boldsymbol{x} \in \mathbb{R}^{18}$ denote the calibrated measurements returned by the data glove, and $\boldsymbol{y} \in \mathbb{R}^{5}$ denote the digit position vector of the DOAs of the hand. The elements in $\boldsymbol{y}$ are ordered as follows: y_1, thumb rotation; y_2, thumb flexion; y_3, index flexion; y_4, middle flexion; y_5, ring/little flexion. The calibrated data glove measurements are then mapped onto robotic digit positions via a linear mapping:

$$\boldsymbol{y} = \boldsymbol{A}\boldsymbol{x}. \tag{S1}$$

The transformation matrix $\boldsymbol{A}$ was selected as follows:

$$\boldsymbol{A}^{\top} = \begin{bmatrix} 0.639 & 0 & 0 & 0 & 0 \\ 0.383 & 0 & 0 & 0 & 0 \\ 0 & 1 & 0 & 0 & 0 \\ -0.639 & 0 & 0 & 0 & 0 \\ 0 & 0 & 0.4 & 0 & 0 \\ 0 & 0 & 0.6 & 0 & 0 \\ 0 & 0 & 0 & 0.4 & 0 \\ 0 & 0 & 0 & 0.6 & 0 \\ 0 & 0 & 0 & 0 & 0 \\ 0 & 0 & 0 & 0 & 0.1667 \\ 0 & 0 & 0 & 0 & 0.3333 \\ 0 & 0 & 0 & 0 & 0 \\ 0 & 0 & 0 & 0 & 0.1667 \\ 0 & 0 & 0 & 0 & 0.3333 \\ 0 & 0 & 0 & 0 & 0 \\ 0 & 0 & 0 & 0 & 0 \\ -0.19 & 0 & 0 & 0 & 0 \\ 0 & 0 & 0 & 0 & 0 \\ 0 & 0 & 0 & 0 & 0 \end{bmatrix} \tag{S2}$$

REFERENCES

Steffen, J., Maycock, J., and Ritter, H. (2011). Robust dataglove mapping for recording human hand postures. In *Proc. Int. Conf. Intell. Robots Appl.* (Springer), 34–45

Wang, Y. and Neff, M. (2013). Data-driven glove calibration for hand motion capture. In *Proc. ACM SIGGRAPH/Eurographics Symp. Comp. Anim.* (ACM Press), 15
